# Observations on the Dangerous Tendency of Dr. Kinglake' Hypothesis, Respecting the Use of Cold Water in Gouty and Rheumatic Affections: Illustrated with a Case, in Reply to a Letter Addressed to Dr. Kinglake, in the London Medical and Physical Journal

**Published:** 1816-12

**Authors:** 


*Observations on the dangerous Tendency of Dr. Kinglake' Hypothesis, respecting the Use of Cold Water in Gouty and Rheumatic Affections; Illustrated with a Case, in reply to a Letter addressed to Dr. Kinglake, in the London Medical and Physical Journal;* by WILLIAM NORMAN, M. R. C. S. Taunton, 1816. 8vo. pp. 53.

IN our review of Dr. Parry's work, we pointed out that gentleman's opinions on the subject of gout, as forming a tolerable contrast with the rash ravings of Dr. Kinglake. We also alluded to the bullying attacks of the latter, on all who dared to publish the fatal effects of the cold water system. Mr. Norman is among the objects of the Doctor's fury, and he has here published a pamphlet, containing some of Dr. K's lucubrations in the Medical and Physical Journal; his own reply to them, and a series of critiques from various periodical and other works, shewing the sense of the medical world, on the subject of the cold water treatment. From his own reply, which is the only original, and previously unpublished part of the pamphlet, we shall select a passage or two, to shew Mr. Norman's style and manner. We are of opinion, however, that Mr. Norman has fallen a little into the error of his antagonist, in adopting that virulence of remark, which so characterises the Doctor's Philippics. The hypothesis and the practice are going fast to the land of oblivion, by their own Lethean tendency, and therefore it was hardly worth the time and labour to refute them seriously.

" The questions at issue are — *are not* gouty and rheumatic inflammation, (not inflammation arising from visible or mechanical causes, as from fractures, sprains, &c. and unconnected with a previous internal disturbance of the system) " migrating" in their qualities? *Do they not* at one time, " station their violence" on one part, at another time on another part? *Do they not* now invade (for instance) the knees? and will they not subsequently attack the feet, the wrists, the elbows; in fine, every part liable to such affections? *Are not* " an undigesting stomach, constipated bowels, and other kind of visceral derangement," invariable precursors of the one, and constant attendants upon both? *Are not* pyrexia, and general inflammatory diathesis of the system, their inseparable associates? *Are not* the external inflammatory appearances on the joints of the extremities, the *effects* of a gouty or rheumatic diathesis previously pervading the habit, *and not the primary cause* of constitutional disturbance? Are there not *other instances* on record, besides the fatal one I have adduced, where by officious interference these salutary efforts have been so interrupted, that translations of the inflammatory action have been almost instantaneously made to the head, chest, or stomach, and the life of the patient thereby sacrificed, or at least greatly endangered? And is it not a notorious fact, that when the disease, after remaining for some time in a joint, ceases entirely, it generally leaves the person in very perfect health, enjoying greater ease and alacrity in the functions both of mind and body, than he had for a long time before experienced? These, and *these only*, are the legitimate points at issue, and not whether (contemptible subterfuge) simple inflammation in its general acceptation possesses "a migrating quality" or not! And if the Doctor can negative these queries upon the testimony of any credible author, living or dead, or if he can do so by the production of facts instead of flippant ipse dixits, by "veritable" demonstrations instead of dreamy speculations, by sound pathological arguments instead of vague "gratuitous" declamation, it his duty so to do — I challenge him to the task, and if he decline it, of what avail has been all that *verbiage* with which he has occupied your pages, compromised his own reputation, and disgraced all scientific disquisition? p. 21

"I will venture to assert, that general experience and the highest medical authorities, have established that gouty and rheumatic inflammation decidedly possess the "fugitive property" of translating their attacks from one part to another; that they are invariably accompanied and preceded by general constitutional disorder; and that if the progress of the external local affections be violently interrupted before the completion of the salutary purposes which they are destined to effect, there is the utmost hazard that some internal organ, already predisposed, may become suddenly oppressed with an increased morbid action, that must imminently endanger, and perhaps rapidly destroy, the existence of the patients. Dr. Kinglake, alone denies these positions; but upon what ground? From his superior pathological knowledge? NO for Dr. Kinglake asserts, that gout does not differ from simple in; flammation arising from external injury, although common experience shows, that simple inflammation, under certain treatment, almost invariably tends to suppuration, and that gouty inflammation under the same treatment, has never been known so to terminate. It is equally well known, that *gouty inflammation* in joints will often instantaneously disappear from the sudden application of cold, leaving the limb at once perfectly free from pain, or any external indication of disease. Does *simple inflammation*, under any circumstance, disappear so rapidly, so completely? It does not demand professional knowledge to answer the question: almost every person from his own experience, can positively reply to it in the negative. *Gouty inflammation*, then, differs from *simple inflammation* in two of its most obvious characteristics. These states of disease neither advance nor retreat in the same manner; and yet Dr Kinglake affirms, they are not only alike but the same — and why? because both are hot and red. — Excellent argument; how like that of Captain Fluellin! "If you look in the maps of the

'orld, I warrant, you shall find in the comparisons between Macedon and Monmouth, that the situations, look you, is both alike. There is a river in Macedon ; and there is moreover a river at Monmouth: it is called Wye, at Monmouth: but it is out of my prains what is the name of the other river ! but *'tis all one, 'tis so like as my fingers is to my fingers, and there is salmons in both.*" I will not speak of the other obvious pathological differences which exist between these two states of disease. What I have adduced is sufficient to prove, that the Doctor must either be ignorant of the symptoms which characterize simple inflammation, on the knowledge of which I believe it will be admitted, that almost the whole superstructure of medical science is raised, or if he be acquainted with them, that he has purposely concealed that knowledge in his book, lest its disclosure should overturn his hypothesis." p. 23.

" Ignorant of pathology and chemistry, as far as those sciences are connected with his subject, or not daring to expose his knowledge of those sciences if he really possessed it. What strange perversion of intellect could induce the doctor, to publish a book in open defiance of the most learned opinions of the age? What still more strange infatuation could lead him to propose his crudities so arrogantly, or to defend them with such disgraceful acrimony ? Could he possibly fancy that he might beguile his readers by the allurements of his style of composition ? The Doctor should have reflected, that the medical world, at least, are not easily misled by mere sound. Pompous obscurity ; the *ignotum pro magnifico* might impose upon the vulgar, but the well informed public have long been convinced, that obscurity in prose writing, forms no part of the sublime—that those who think clearly can always express their thoughts intelligibly ; and that no accumulation of big words can be taken as an equivalent for deficiency of meaning." p. 25.

We need not extract any more specimens of Mr. Norman's manner or matter. Dr. Kinglake has in Mr. N. roused an antagonist, who seems both able and willing to fight him with his own weapons ; but more polished and acute. We are disposed to think, that if the Doctor *feels* at all, he will not sit very easy under the shaving process which Messrs. Ring and Norman have instituted on their Taunton friend.

For our own parts, we have not ceased to expose the pernicious tendency, both of the repellent and expellent systems in gout. Fresh instances of their fatality will, ere long, be communicated in this Journal ; and we invite the friends of science and humanity, to furnish us with such facts as have come to their knowledge on this point, in order that the practice may, as soon as possible, be banished from the medical world.

